# Sex and Age-Dependent Olfactory Memory Dysfunction in ADHD Model Mice

**DOI:** 10.3390/life13030686

**Published:** 2023-03-02

**Authors:** Jae-Sang Sim, Won-Seok Lee, Bo-Eun Yoon

**Affiliations:** Department of Molecular Biology, Dankook University, Cheonan 31116, Republic of Korea

**Keywords:** ADHD, GIT1, neural stem cell, olfactory memory

## Abstract

ADHD is a typical neurodevelopmental disorder with a high prevalence rate. NSCs in the subventricular zone (SVZ) are closely related to neurodevelopmental disorder and can affect olfactory function by neurogenesis and migratory route. Although olfactory dysfunction is one of the symptoms of ADHD, the relevance of cells in the olfactory bulb derived from NSCs has not been studied. Therefore, we investigated olfactory memory and NSCs in Git1-deficient mice, under the ADHD model. Interestingly, only adult male G protein-coupled receptor kinase-interacting protein-1 (GIT1)-deficient (+/−, HE) mice showed impaired olfactory memory, suggesting sex and age dependence. We performed adult NSCs culture from the SVZ and observed distinct cell population in both sex and genotype. Taken together, our study suggests that the altered differentiation of NSCs in GIT1+/− mice can contribute to olfactory dysfunction in ADHD.

## 1. Introduction

ADHD is a neurodevelopmental disorder that can present symptoms at all ages and is characterized by attention deficit, hyperactivity, and impulsivity [1] There are sex differences in ADHD prevalence [2]. The ratio is 2:1 and 1.6:1 in childhood and adulthood, respectively [3]. These sex differences in symptoms have also been found in patients, and in the case of males, external symptoms such as hyperactivity and impulsivity predominated. The symptoms of female patients were prominent internal symptoms such as inattention, low self-esteem, and anxiety [4]. Therefore, to establish a research strategy for ADHD in both sexes, we have to understand the different symptoms and mechanisms between males and females.

Olfactory dysfunction is one of the symptoms of ADHD [5]. Many studies reported that the ratio of males having olfactory dysfunction was higher than females among ADHD patients [6]. Because the olfactory bulb (OB) performs olfactory discrimination, identification, learning, and memory, alteration of OB cells can affect olfactory behavior [6]. Especially, the generation of adult-born neurons is essential for olfactory behavior and is grouped into two cell types: granule cells (GCs) and periglomerular cells (PGCs) in the OB [7]. In OB cell populations, PGCs are GABA- or dopaminergic [8,9], and GCs are only GABAergic neurons [10,11]. Adult-born interneurons are continuously replaced by NSCs and perform inhibitory modulation in the olfactory circuit via GABAergic events [12]. This inhibitory modulation is regulated by tonic inhibition [13]. Additionally, adult-born neurons affect olfactory behavior, such as discrimination [14]. Accordingly, it is necessary to research olfactory neurogenesis to study olfactory behavior. The origin of newborn cells in the OB is NSCs in the subventricular zone (SVZ) [6]. NSCs can self-renew and differentiate neurons, astrocytes, and oligodendrocytes [15]. The NSCs migrate from the SVZ to the OB through the rostral migratory stream (RMS) pathway [16,17]. The RMS is mainly composed of immature neurons from the SVZ. In the OB, immature neurons are differentiated into interneurons and integrated into existing olfactory circuits [18,19,20,21,22]. For the reason above, the OB can be affected by the potency and migration of SVZ-derived NSCs. Increased proliferation of SVZ-NSCs leads to the upregulation of olfactory neurogenesis [23,24,25,26] and olfactory discrimination [27]. Similarly, increased immature neurons in the SVZ improve olfactory discrimination [28] and memory performance [29].

The causes of ADHD onset are heredity, low birth weight, fetal alcohol spectrum disorders, etc. [30]. According to twin studies, it seems to be caused by genetics, with a high probability of 70~80% in childhood and 44% in adulthood [31,32]. GIT1 is one of the genes related to the prevalence of ADHD in Korean childhood [33]. Since GIT1 is an adaptor protein with several protein domains, it is involved in various signaling pathways [34,35,36]. In the Intron 20 of the GIT1 gene, single nucleotide polymorphism (SNP) that replaces allele C by T increases ADHD risk. This SNP caused a reduction of GIT1 expression. GIT1-deficient mice showed ADHD symptoms such as hyperactivity and impaired memory, which were normalized by amphetamine, a drug to ameliorate ADHD [33]. GIT1 is related to diverse neurodevelopmental processes. For example, GIT1 regulates neuronal spine morphogenesis, neurite extension, and synapse formation [37,38,39,40,41,42] and is also essential in astrogenesis [43]. Therefore, we used GIT1 +/− mice for this study. Using ADHD mice, we aimed to determine if olfactory dysfunction was sex-specific and study the reasons for this symptom in the cell population generated by NSCs from the SVZ.

## 2. Materials and Methods

### 2.1. Animal

GIT1 mice were crossbred 129S1/SvlmJ and C57BL/6. Possible genotypes are GIT1 wild-type (+/+, WT), used to control mice, GIT1 heterozygous type (+/−, HE), and GIT1 knock-out (−/−, KO). Genotyping of GIT1 mice was performed by Polymerase chain reaction (PCR) of Deoxyribonucleic acid (DNA) from the tail-tip. Part of the GIT1 sequence was used for GIT1+/+ primer and the beta-galactosidase sequence was used for GIT1−/− primer. All the animal experiments described below were performed in accordance with Dankook University Animal Experimentation Guidelines (approval number DKU-19–016, Cheonan, Republic of Korea).

### 2.2. Primary Neural Stem Cell Culture

Primary NSC cultures were performed using previously published methods [44]. Medium for NSC culture was DMEM/F12 (with 15 mM HEPES and L-glutamine, cat #LM002-04, Welgene, Gyeongsan-si, Republic of Korea) composed of 2% B27 supplement (without vitamin A, cat #12587-010, Gibco, Billings, MT, USA), 2 μg/mL heparin (cat #07980, STEMCELL, Vancouver, British Columbia, Canada), 1× GlutaMAXTM supplement (cat #35050061, Gibco), and 100 unit/mL penicillin/streptomycin (cat #LS202-02, Welgene). Tissue from the subventricular zone was minced using a sterile blade for 1 min. The tissue was dissociated using 1 mL StemProTM AcuutaseTM (cat# A1110501, Gibco) included 0.125 mg/mL DNase I (cat #11284932001, Roche, Basel, Switzerland) and incubated for 7 min at 37 °C. Dissociated tissue was centrifuged for 5 min at 300× *g* (High-Speed Centrifuges, MGR1580, Gyrozen, Gimpo, Republic of Korea) and the supernatant was removed. We resuspended the pellet with 1 mL medium and added 4 mL medium. 5 mL medium was sieved through a 40 μm cell strainer (cat #93040, SPL, Seattle, WA, USA) and centrifuged for 5 min at 300× *g*. the pellet was resuspended by 200 μL growth medium containing two growth factors, 20 ng/mL FGF-2 (cat #GF003, Merck, Rahway, NJ, USA) and EGF (cat #GF144, Merck). Lastly, we diluted cell suspension to 20 mL growth medium and plate 200 μL/well across a 96-well plate (cat #30096, SPL). A cell plate was incubated for 7 days at 37 °C with 5% CO_2_. After 7 days from neural stem cell culture, primary neurospheres were collected in a 15 mL tube (cat #50015, SPL) and centrifuged for 5 min at 300× *g*. To dissociate to single cells, neurospheres were resuspended by 1 mL StemProTM AccutaseTM and incubated for 5 min at room temperature. Adding 4 mL 1 × PBS, a total of 5 mL cell suspension was centrifuged for 5 min at 300× *g*. Cells were resuspended by 1 mL medium and plated onto a 24-well cell floater plate (cat #39724, SPL) at 1 × 104 cells/mL density. To adhere neural stem cells on a plate (or coverslip), we first added enough poly-d-lysine (PDL) (10 μg/mL in DW, cat #354210, Corning, Somerville, MA, USA) solution and incubated it overnight at room temperature. The PDL was removed, and the plate was washed with DW 3 times and allowed to air dry. After drying, Laminin (5 μg/mL in cold DMEM/F12, cat #23017015, Gibco) solution was added to the PDL-coated plate, and the plate was incubated overnight at 37 °C.

### 2.3. Differentiation of Neural Stem Cells

Neural stem cells were dispensed to the coated plate in the presence of media with 10 ng/mL FGF-2 and 20 ng/mL EGF. After 2 days, the media was replaced with 5 ng/mL FGF-2. After 2 days again, cell cultures were incubated for 3 days in the presence of media without growth factors.

### 2.4. Immunocytochemistry

Cells were differentiated on a coated coverslip in a 24-well plate (cat #330024, SPL) and were washed 1 × PBS for 5 min 3 times. Washed cells were fixed with 4% paraformaldehyde (PFA) for 20 min at room temperature. After fixation, cells were washed 1 × PBS for 5 min 3 times and incubated in blocking solution (1 × PBS with 2% normal goat serum (cat #005-00-121, Jackson Immuno Research Inc., Philadelphia, PA, USA) and 0.3% Triton X-100 (cat #X-100, Sigma, St. Louis, MO, USA)) for 1 h at room temperature on a shaker. The cells were incubated in the presence of primary antibody overnight at 4 °C on a shaker. Used primary antibodies and the titer were as follows. Chicken anti-MAP2 (cat #ab5392, Abcam, Cambridge, UK) for a neuronal marker protein was diluted 1:1000 with a blocking solution. Rabbit anti-S100beta (cat #ab52642, Abcam) for astrocytic marker protein and Mouse anti-O4 (cat# MAB1326, R&D systems, Minneapolis, MN, USA) for oligodendrocyte marker protein were diluted 1:500 with blocking solution. At the end of the primary antibody incubation, cells were washed with 1 × PBS for 5 min 3 times and incubated in the presence of secondary antibody for 1 h at room temperature on a shaker. The used secondary antibody and the titer were as follows. Alexa 488-conjugated goat anti-chicken antibody (cat #103-545-155, Jackson Immuno Reseach Inc.), Alexa 488-conjugated goat anti-mouse antibody (cat #115-545-003, Jackson Immuno Reseach Inc.), and Alexa 594-conjugated goat anti-rabbit antibody (cat #111-585-003, Jackson Immuno Reseach Inc.) were diluted 1:1000. After secondary antibody incubation, cells were washed with 1 × PBS for 5 min 2 times and incubated in 1 × PBS with DAPI (cat #d9542, Sigma), the titer was 1:1000 for 5 min at room temperature. After the last washing for 5 min, the coverslip was overturned on Faramount Mounting Medium (cat #S3025, Dako, Carpinteria, CA, USA) on slide glass (cat #S7441, Matsunami, Bellingham, WA, USA) and allowed to air dry. The completed sample was stored at −80 °C. Imaging and analysis were performed using a confocal microscope (Zeiss LSM700, Dublin, CA, USA) and Image J software, respectively.

### 2.5. Immunohistochemistry

#### Cryocut

To fix the mouse brain tissue, separated tissue was incubated in 4% PFA overnight at 4 °C. After fixation, tissue was washed with 1 × PBS for 5 min 3 times. To drain water, tissue was left in the 10 mL of sucrose solution (30% in 1 × PBS) by sinking to the bottom for about 2 days. The tissue was frozen at −80 °C in the presence of the O.C.T. compound (cat #4583, Tissue-Tek, Torrance, CA, USA). Tissue slices were acquired by using cryocut (CM3050S, Leica, Wetzlar, Germany), and the thickness was 30 μm and 50 μm into coronal and sagittal sections, respectively. Acquired tissues were stored in a tissue storage buffer consisting of 10% 1 × PBS (pH 7.2), 30% Glycerol, 30% Ethylene glycol, and 30% DW at −20 °C.

### 2.6. 3,3′-Diaminobenzidine (DAB) Staining

The tissue slice was washed with 1 × PBS-T (0.2% Triton X-100) for 10 min 2 times and then left in 3% hydrogen peroxide (H_2_O_2_) solution (cat #H1009, Sigma) for 4 min and 30 s. The tissue slice was additionally washed for 10 min 3 times and incubated in primary antibody-diluted 1 × PBS-T overnight at 4 °C on a shaker. The primary antibody was rabbit anti-doublecortin (cat #ab18723, Abcam) and the titer was 1:2000. The next day, washing was performed for 10 min 3 times. The tissue slice was incubated with a secondary antibody for 1 h at room temperature on a shaker. The secondary antibody was goat anti-rabbit (Biotinylated) (cat #BA-1000-1.5, Vector Laboratories, Newark, CA, USA). After secondary antibody incubation, the tissue slice was washed for 10 min 3 times and incubated for 1 h at room temperature using VECTASTAIN^®^ ABC-HRP Kit (cat #PK-4000, Vector Laboratories). After ABC incubation, washing was performed for 10 min 3 times. To perform colorimetric development, the tissue slice was incubated in 0.1M DAB solution with 1/100 diluted 30% H_2_O_2_ for 45 s and washed once. And then tissue slice was allowed to air dry on slide glass. The dried sample was dehydrated for 10 min at each step using gradual ethanol of 70 to 100% concentration. The dehydrated sample was left in xylene overnight. Lastly, the sample was covered with cover glass in the presence of permount mounting solution (cat #SP15-100, Fisher Chemical, Waltham, MA, USA) and allowed to air dry. Imaging and analysis were performed by using an optical microscope and ImageJ software, respectively. DAB-color development was calculated through the following equation as optical density.

ptical Density=log(maxintensity / mean intensity) 

### 2.7. Olfactory Memory Test

Mice were habituated to test cages in the experimental room for 30 min. First, baseline sniff time was time to sniff towards a dish containing the new odor and measured for 5 min at the training phase. Second, the sniffing time same odor as the training phase was measured during the recall phase. Then, ‘%baseline sniff time’ was calculated as the percentage of sniffing time at the recall phase to baseline sniff time. Intervals between the training phase and the recall phase were 0.5, 2, 4, 6, and 24 h. The period between the recall phase and the next training phase was more than 24 h. Treatment of selegiline was performed at a concentration of 10 mg/kg/day in the water.

### 2.8. Statistics

Statistical analysis was achieved by using Microsoft Excel and GraphPad Prism 9. Statistical significance was evaluated through a two-tailed unpaired *t*-test and a one-way ANOVA test. Numerical data were presented as ‘average ± SEM.’ * *p* < 0.05, ** *p* < 0.01, *** *p* < 0.001.

## 3. Results

### 3.1. Impaired Olfactory Memory in GIT1+/− Mice

**Figure 1:**
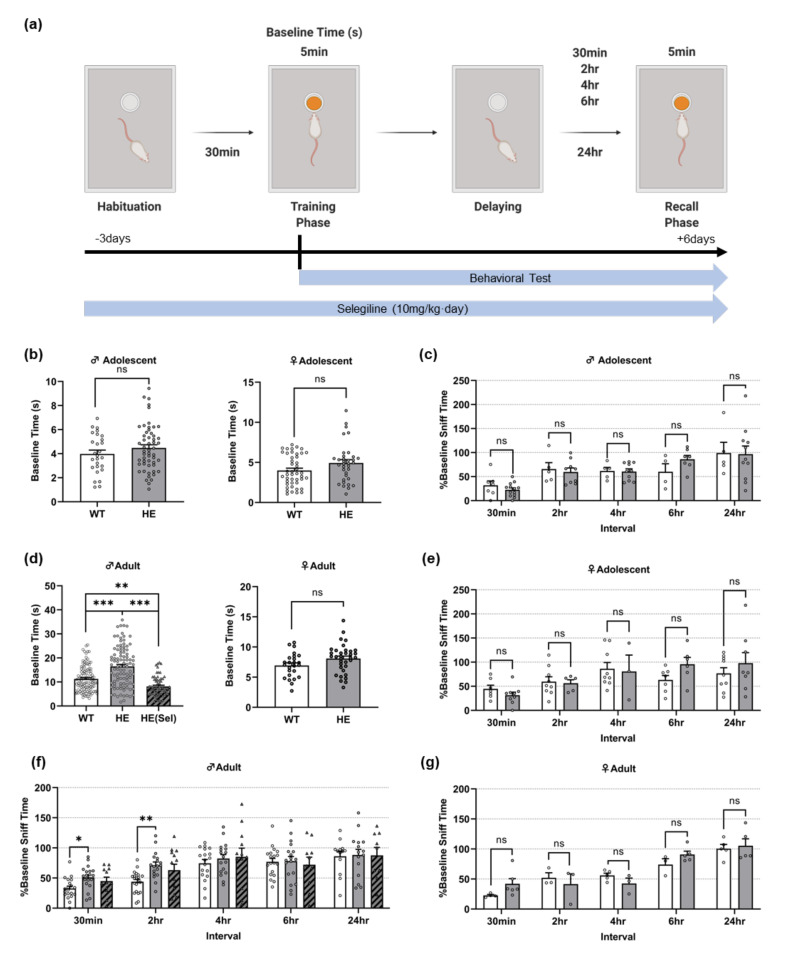
(**a**) Scheme of behavioral experiment of olfactory memory test. Baseline time and sniff time was measured at training phase and recall phase, respectively. The arrow below shows the timeline for the behavior test and selegiline administration. (**b**,**d**) are baseline time of sniffing in training phase for adolescent (**b**) males (**left**) and females (**right**), and adult (**d**) males (**left**) and females (**right**). (**c**,**e**–**g**) Percentage of sniffing time in recall phase about training phase depending on interval. The graphs indicated the percentage of sniffing times of adolescent males (**c**) and females (**d**), and adult males (**f**) and females (**g**). * *p* < 0.1, ** *p* < 0.01, *** *p* < 0.001, “ns”—non-significant.

We performed the olfactory memory test to determine whether GIT1+/− mice exhibit impaired olfactory memory, one of the various ADHD symptoms [5] (Figure 1a). In the case of adolescents, there was no difference in sniffing time during the training phase (baseline time) between the GIT1+/+ and GIT1+/− mice, regardless of sex (Figure 1b). Olfactory memory was also no different between GIT1+/+ mice and GIT1+/− mice (Figure 1c,e). However, we confirmed that adult male GIT1+/− mice had increased baseline time compared with GIT1+/+ mice (Figure 1d). Notably, the olfactory memory of male GIT1+/− mice showed lower performance at intervals 30 min and 2 h of the test phase than GIT1+/+ mice (Figure 1f). Moreover, we found that the impairment in olfactory memory was reversed when GIT1+/− male mice were given selegiline, which affects treatment for ADHD [45] (Figure 1d left,f). However, female GIT1+/− mice had no difference in olfactory memory compared with GIT1+/+ mice, indicating a sex difference. (Figure 1g). These results suggest that only adult male GIT1+/− mice, not female mice, had olfactory dysfunction.

### 3.2. Contribution of Migrating Immature Neurons on RMS to Olfactory Neuronal Population

**Figure 2:**
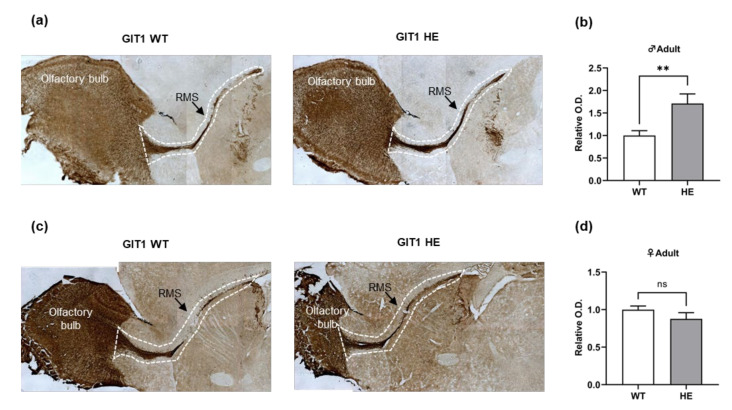
Doublecortin intensity of immature neuron in rostral migratory stream. (**a**,**c**) Representative images of DAB staining for doublecortin in rostral migratory stream from adult GIT1 male and female mice. (**b**,**d**) Relative optical density of doublecortin in rostral migratory stream from adult GIT1 male and female mice. Optical density was analyzed in region of interest (ROI; white dashed line). ** *p* < 0.01, “ns”—non-significant.

We assessed the migration of immature neurons from the SVZ to the OB to investigate whether the differentiation of NSCs in the SVZ reflects cells in the OB. Therefore, we used DAB staining to label the RMS with doubletin (DCX), a marker of immature neurons, and analyzed the intensity of DCX. In adult male mice, the DCX intensity of GIT1+/− mice was significantly higher than GIT1+/+ mice (Figure 2a,b). However, in adult female mice, the DCX intensity of GIT1+/− mice was no different from GIT1+/+ mice (Figure 2c,d). Therefore, we confirmed that the number of immature neurons migrating to the OB increased in GIT1+/− male mice, indicating that the differentiation of NSCs into mature neurons was reduced in the SVZ.

### 3.3. Decreased Neuronal Differentiation of GIT1+/− Neural Stem Cells

**Figure 3:**
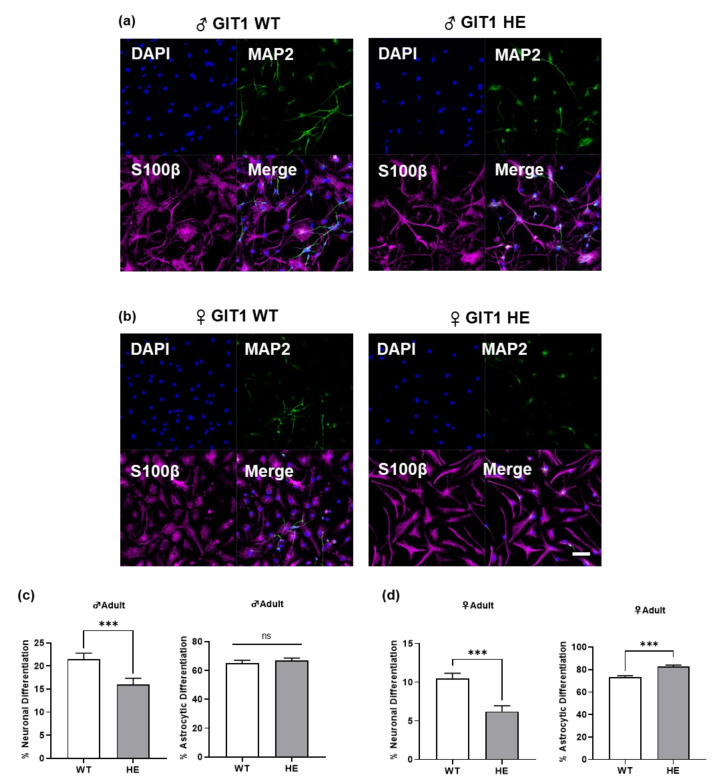
Differentiation of subventricular zone neural stem cells in adult GIT1 mice. (**a**,**b**) Representative images of immunocytochemistry in differentiation of neural stem cells from adult GIT1 male and female mice (left-WT, right-HE/blue-DAPI, green-MAP2, magenta-S100β). (**c**,**d**) Percentage of differentiation from neural stem cell to neuron (**left**) and astrocyte (**right**), scale bar = 50 μm. *** *p* < 0.001, “ns”—non-significant.

Since cell populations in the OB are known to be derived from NSCs in the SVZ [16,17], we cultured NSCs from the SVZ in GIT1 mice and induced cells to differentiate into neurons and astrocytes. We stained the cells using neuron and astrocyte markers to confirm which type of cells they differentiated into. In the adult stage, we observed that the NSCs of GIT1+/− male mice showed lower neuronal differentiation than GIT1+/+ mice (Figure 3a,c left). However, astrocyte differentiation in the male GIT1 mice showed no difference between both genotypes. (Figure 3a,c right). Similar to the male data, we found that the differentiation of NSCs into neurons in female GIT1+/− mice was diminished compared to GIT1+/+ mice (Figure 3b,d left). In contrast to male mice, the differentiation of NSCs from female GIT1 +/− mice into astrocytes was reduced (Figure 3b,d right). Altogether, this indicated that neuronal differentiation of NSCs in GIT1+/− was lower than GIT1+/+ regardless of sex, but differentiation into astrocytes of NSCs was affected by a sex difference in vitro.

## 4. Discussion

Impaired olfactory memory is one of the symptoms of ADHD and has been studied in human research [5]. Moreover, previous studies have reported that the proportion of males with olfactory dysfunction in ADHD is higher than females [46]. In addition, a clinical study confirmed through structural magnetic resonance imaging, that the size of the olfactory bulb increased in boys with ADHD [47]. In addition, according to a recent clinical study of sleep-associated olfactory memory in ADHD patients, olfaction might be a biomarker of ADHD [48]. Therefore, we investigated whether olfactory memory dysfunctions appeared in GIT1+/− mice and whether the cell population was altered in the OB. We observed that olfactory memory dysfunctions appeared only in male mice, which is similar to human studies [46]. Furthermore, we identified immature neurons migrating from the SVZ to the OB, which could not be observed in human studies, were increased in only GIT1+/− male mice. In addition, we confirmed that the decreased neuronal differentiation of NSCs in the SVZ only appeared in GIT1+/− male mice, whereas differentiation into astrocytes was increased in GIT^+/−^ female mice in vitro. As our research found, there are many studies on behavior modulated by the neuronal population, which could induce physiological changes in the brain and regulate neuronal circuits [49,50]. Changes in neuronal and glial cell populations in the prefrontal cortex of autism spectrum disorder could be critical anatomical pathogenesis [51]. Therefore, we suggest that the different cell populations in the OB could influence olfactory dysfunction. Moreover, we presumed that sex differences in olfactory function would be due to differently differentiated cell populations according to gender. However, because GIT1+/− mice were not a cell type-specific deficient model, we could not determine the contribution of each cell type [33]. The cellular and behavioral difference between GIT1+/− mice compared to GIT1+/+ mice may have resulted from a complex interaction between multiple cell types such as neurons and glial cells [41,43]. GIT1 is known to regulate neuronal spine morphogenesis, neurite outgrowth, and synapse formation [37,41,42]. In addition, GIT1 deficiency has been associated with abnormal astrocytosis in the basal ganglia [43]. In our previous study, decreased astrocytic GABA and tonic inhibition appeared in the cerebellum of GIT1-deficient mice and could cause impaired motor coordination [52]. A limitation of this study was the use of a single gene mutation animal model. However, the hetero type of GIT1 showed significant association in human SNP study and ADHD-like behavior when this gene was deleted in mice, which confirmed that these symptoms were recovered by methylphenidate, an ADHD treatment drug used in clinical practice [33]. Therefore, it can be one of the representative ADHD animal models. Of course, animal ADHD-like behavior cannot be the same as a human symptom. But it is worth studying because animal testing allows us to quickly determine the effectiveness of drugs on certain symptoms and explore the brain regions and molecular mechanisms involved. The effects of different cell types in the olfactory bulb of the GIT1-deficient mice and a general value in the cell area or size must be further studied. Furthermore, it is necessary to study this using an NSC-specific genetic modification model rather than a whole genome-modified model. It is also essential to study the molecular mechanisms of differentiation differences in NSCs in GIT1 mice that cause changes in the cell population. We suggest that it is necessary to investigate the overall olfactory dysfunction, an ADHD symptom that has not been studied, through additional behavioral experiments.

## 5. Conclusions

In conclusion, we demonstrated that olfactory dysfunction in human ADHD patients was reenacted in GIT1+/− adult male mice. Moreover, we observed a more significant number of mature neurons in the RMS of GIT1+/− male mice. When NSCs from the SVZ of ADHD model mice were cultured and differentiated in vitro, we found that differentiation into neurons was reduced in GIT1+/− mice in both sexes. Astrocyte differentiation was increased in only female mice. Therefore, we propose that cell differentiation for the sex-specific differences could induce distinct olfactory memory performance in the ADHD model. 

## Data Availability

Not applicable.

## Abbreviations

−/−: KO: knock-out; +/−, HE: heterozygous type; +/+, WT: wild-type; ADHD: Attention-Deficit/Hyperactivity Disorder; DAB: 3, 3’-Diaminobenzidine; DCX: doublecortin; DNA: Deoxyribonucleic acid; GCs: granule cells; GIT1: G protein-coupled receptor kinase-interacting protein-1; NSCs: neural stem cells; OB: olfactory bulb; PCR: Polymerase chain reaction; PDL: poly-d-lysine; PGCs: periglomerular cells; RMS: rostral migratory stream; SNP: single nucleotide polymorphism; SVZ: subventricular zone.

## References

[1] Kessler R.C., Adler L., Barkley R., Biederman J., Conners C.K., Demler O., Faraone S.V., Greenhill L.L., Howes M.J., Secnik K. (2006). The Prevalence and Correlates of Adult ADHD in the United States: Results from the National Comorbidity Survey Replication. Am. J. Psychiatry.

[2] Ramtekkar U.P., Reiersen A.M., Todorov A.A., Todd R.D. (2010). Sex and Age Differences in Attention-Deficit/Hyperactivity Disorder Symptoms and Diagnoses: Implications for DSM-V and ICD-11. J. Am. Acad. Child Adolesc. Psychiatry.

[3] Schultz S.K., Kuhl E.A., APA (2013). Diagnostic and Statistical Manual of DSM-5 TM.

[4] Rucklidge J.J. (2010). Gender Differences in Attention-Deficit/Hyperactivity Disorder. Psychiatr. Clin. North Am..

[5] Yilmaz Y., Ince E., Ugurlu H., Bas A., Tatli B., Balcioglu I. (2015). Clinical Assessment and Implication of Olfactory Dysfunction in Neuropsychiatric Disorders of Childhood and Adulthood: A Review of Literature. J. Neurobehav. Sci..

[6] Lim D.A., Alvarez-Buylla A. (2016). The Adult Ventricular–Subventricular Zone (V-SVZ) and Olfactory Bulb (OB) Neurogenesis. Cold Spring Harb. Perspect. Biol..

[7] Bagley J., LaRocca G., Jimenez D.A., Urban N.N. (2007). Adult Neurogenesis and Specific Replacement of Interneuron Subtypes in the Mouse Main Olfactory Bulb. BMC Neurosci..

[8] Kosaka K., Kosaka T. (2007). Chemical Properties of Type 1 and Type 2 Periglomerular Cells in the Mouse Olfactory Bulb Are Different from Those in the Rat Olfactory Bulb. Brain Res..

[9] Parrish-Aungst S., Shipley M.T., Erdelyi F., Szabo G., Puche A.C. (2007). Quantitative Analysis of Neuronal Diversity in the Mouse Olfactory Bulb. J. Comp. Neurol..

[10] Mori K., Kishi K., Ojima H. (1983). Distribution of Dendrites of Mitral, Displaced Mitral, Tufted, and Granule Cells in the Rabbit Olfactory Bulb. J. Comp. Neurol..

[11] Orona E., Scott J.W., Rainer E.C. (1983). Different Granule Cell Populations Innervate Superficial and Deep Regions of the External Plexiform Layer in Rat Olfactory Bulb. J. Comp. Neurol..

[12] Lledo P.-M., Saghatelyan A., Lemasson M. (2004). Inhibitory Interneurons in the Olfactory Bulb: From Development to Function. Neuroscience.

[13] Labarrera C., London M., Angelo K. (2013). Tonic Inhibition Sets the State of Excitability in Olfactory Bulb Granule Cells. J. Physiol..

[14] Gheusi G., Cremer H., McLean H., Chazal G., Vincent J.-D., Lledo P.-M. (2000). Importance of Newly Generated Neurons in the Adult Olfactory Bulb for Odor Discrimination. Proc. Natl. Acad. Sci. USA.

[15] De Filippis L., Binda E. (2012). Concise Review: Self-Renewal in the Central Nervous System: Neural Stem Cells from Embryo to Adult. Stem Cells Transl. Med..

[16] Doetsch F., Alvarez-Buylla A. (1996). Network of Tangential Pathways for Neuronal Migration in Adult Mammalian Brain (Subventricular Zonesubependymal Layerneurogenesis). Proc. Natl. Acad. Sci. USA.

[17] Lois C., García-Verdugo J.-M., Alvarez-Buylla A. (1996). Chain Migration of Neuronal Precursors. Science.

[18] Altman J. (1969). Autoradiographic and Histological Studies of Postnatal Neurogenesis IV. Cell Proliferation and Migration in the Anterior Forebrain, with Special Reference to Persisting Neurogenesis in the Olfactory Bulb. J. Comp. Neurol..

[19] Lois C., Alvarez-Buylla A. (1994). Long-Distance Neuronal Migration in the Adult Mammalian Brain. Science.

[20] Kornack D.R., Rakic P. (2001). The Generation, Migration, and Differentiation of Olfactory Neurons in the Adult Primate Brain. Proc. Natl. Acad. Sci. USA.

[21] Pencea V., Bingaman K.D., Freedman L.J., Luskin M.B. (2001). Neurogenesis in the Subventricular Zone and Rostral Migratory Stream of the Neonatal and Adult Primate Forebrain. Exp. Neurol..

[22] Defteralı Ç., Moreno-Estellés M., Crespo C., Díaz-Guerra E., Díaz-Moreno M., Vergaño-Vera E., Nieto-Estévez V., Hurtado-Chong A., Consiglio A., Mira H. (2021). Neural Stem Cells in the Adult Olfactory Bulb Core Generate Mature Neurons in Vivo. Stem Cells.

[23] Zigova T., Pencea V., Wiegand S.J., Luskin M.B. (1998). Intraventricular Administration of BDNF Increases the Number of Newly Generated Neurons in the Adult Olfactory Bulb. Mol. Cell. Neurosci..

[24] Gómez-Gaviro M.V., Scott C.E., Sesay A.K., Matheu A., Booth S., Galichet C., Lovell-Badge R. (2012). Betacellulin Promotes Cell Proliferation in the Neural Stem Cell Niche and Stimulates Neurogenesis. Proc. Natl. Acad. Sci. USA.

[25] Machold R., Hayashi S., Rutlin M., Muzumdar M.D., Nery S., Corbin J.G., Dudek H., Mcmahon A.P. (2003). Sonic Hedgehog Is Required for Progenitor Cell Maintenance in Telencephalic Stem Cell Niches.

[26] Adachi K., Mirzadeh Z., Sakaguchi M., Yamashita T., Nikolcheva T., Gotoh Y., Peltz G., Gong L., Kawase T., Alvarez-Buylla A. (2007). β-Catenin Signaling Promotes Proliferation of Progenitor Cells in the Adult Mouse Subventricular Zone. Stem Cells.

[27] Bragado Alonso S., Reinert J.K., Marichal N., Massalini S., Berninger B., Kuner T., Calegari F. (2019). An Increase in Neural Stem Cells and Olfactory Bulb Adult Neurogenesis Improves Discrimination of Highly Similar Odorants. EMBO J..

[28] Mishra S.K., Hidau M. (2021). Intranasal Insulin Enhances Intracerebroventricular Streptozotocin–Induced Decrease in Olfactory Discriminative Learning via Upregulation of Subventricular Zone–Olfactory Bulb Neurogenesis in the Rat Model. Mol. Neurobiol..

[29] Mastrodonato A., Barbati S.A., Leone L., Colussi C., Gironi K., Rinaudo M., Piacentini R., Denny C.A., Grassi C. (2018). Olfactory Memory Is Enhanced in Mice Exposed to Extremely Low-Frequency Electromagnetic Fields via Wnt/β-Catenin Dependent Modulation of Subventricular Zone Neurogenesis. Sci. Rep..

[30] Nigg J.T. (2006). What Causes ADHD?.

[31] Nikolas M.A., Burt S.A. (2010). Genetic and Environmental Influences on ADHD Symptom Dimensions of Inattention and Hyperactivity: A Meta-Analysis. J. Abnorm. Psychol..

[32] Reiersen A.M., Constantino J.N., Grimmer M., Martin N.G., Todd R.D. (2008). Evidence for Shared Genetic Influences on Self-Reported ADHD and Autistic Symptoms in Young Adult Australian Twins. Twin Res. Hum. Genet..

[33] Won H., Mah W., Kim E., Kim J.W., Hahm E.K., Kim M.H., Cho S., Kim J., Jang H., Cho S.C. (2011). GIT1 Is Associated with ADHD in Humans and ADHD-like Behaviors in Mice. Nat. Med..

[34] Premont R.T., Claing A., Vitale N., Freeman J.L.R., Pitcher J.A., Patton W.A., Moss J., Vaughan M., Lefkowitz R.J. (1998). β _2_ -Adrenergic Receptor Regulation by GIT1, a G Protein-Coupled Receptor Kinase-Associated ADP Ribosylation Factor GTPase-Activating Protein. Proc. Natl. Acad. Sci. USA.

[35] Claing A., Perry S.J., Achiriloaie M., Walker J.K.L., Albanesi J.P., Lefkowitz R.J., Premont R.T. (2000). Multiple Endocytic Pathways of G Protein-Coupled Receptors Delineated by GIT1 Sensitivity. Proc. Natl. Acad. Sci. USA.

[36] Hoefen R.J., Berk B.C. (2006). The Multifunctional GIT Family of Proteins. J. Cell Sci..

[37] Za L., Albertinazzi C., Paris S., Gagliani M., Tacchetti C., de Curtis I. (2006). ΒPIX Controls Cell Motility and Neurite Extension by Regulating the Distribution of GIT1. J. Cell Sci..

[38] Menon P., Deane R., Sagare A., Lane S.M., Zarcone T.J., O’Dell M.R., Yan C., Zlokovic B.V., Berk B.C. (2010). Impaired Spine Formation and Learning in GPCR Kinase 2 Interacting Protein-1 (GIT1) Knockout Mice. Brain Res..

[39] Zhang H., Webb D.J., Asmussen H., Niu S., Horwitz A.F. (2005). A GIT1/PIX/Rac/PAK Signaling Module Regulates Spine Morphogenesis and Synapse Formation through MLC. J. Neurosci..

[40] Segura I., Essmann C.L., Weinges S., Acker-Palmer A. (2007). Grb4 and GIT1 Transduce EphrinB Reverse Signals Modulating Spine Morphogenesis and Synapse Formation. Nat. Neurosci..

[41] Zhang H., Webb D.J., Asmussen H., Horwitz A.F. (2003). Synapse Formation Is Regulated by the Signaling Adaptor GIT1. J. Cell Biol..

[42] Ko J., Kim S., Valtschanoff J.G., Shin H., Lee J.-R., Sheng M., Premont R.T., Weinberg R.J., Kim E. (2003). Interaction between Liprin-and GIT1 Is Required for AMPA Receptor Targeting. J. Neurosci..

[43] Lim S.Y., Mah W. (2015). Abnormal Astrocytosis in the Basal Ganglia Pathway of Git1−/− Mice. Mol. Cells.

[44] Walker T.L., Kempermann G. (2014). One Mouse, Two Cultures: Isolation and Culture of Adult Neural Stem Cells from the Two Neurogenic Zones of Individual Mice. J. Vis. Exp..

[45] Akhondzadeh S., Tavakolian R., Davari-Ashtiani R., Arabgol F., Amini H. (2003). Selegiline in the Treatment of Attention Deficit Hyperactivity Disorder in Children: A Double Blind and Randomized Trial. Prog. Neuropsychopharmacol. Biol. Psychiatry.

[46] Crow A.J.D., Janssen J.M., Vickers K.L., Parish-Morris J., Moberg P.J., Roalf D.R. (2020). Olfactory Dysfunction in Neurodevelopmental Disorders: A Meta-Analytic Review of Autism Spectrum Disorders, Attention Deficit/Hyperactivity Disorder and Obsessive–Compulsive Disorder. J. Autism. Dev. Disord..

[47] Lorenzen A., Scholz-Hehn D., Wiesner C.D., Wolff S., Bergmann T.O., van Eimeren T., Lentfer L., Baving L., Prehn-Kristensen A. (2016). Chemosensory Processing in Children with Attention-Deficit/Hyperactivity Disorder. J. Psychiatr. Res..

[48] Munz M., Wiesner C.D., Vollersen-Krekiehn M., Baving L., Prehn-Kristensen A. (2022). Sleep Fosters Odor Recognition in Children with Attention Deficit Hyperactivity Disorder but Not in Typically Developing Children. Brain Sci..

[49] Allen W.E., Chen M.Z., Pichamoorthy N., Tien R.H., Pachitariu M., Luo L., Deisseroth K. (2019). Thirst Regulates Motivated Behavior through Modulation of Brainwide Neural Population Dynamics. Science.

[50] Chen J.L., Andermann M.L., Keck T., Xu N.L., Ziv Y. (2013). Imaging Neuronal Populations in Behaving Rodents: Paradigms for Studying Neural Circuits Underlying Behavior in the Mammalian Cortex. J. Neurosci..

[51] Falcone C., Mevises N.Y., Hong T., Dufour B., Chen X., Noctor S.C., Martínez Cerdeño V. (2021). Neuronal and Glial Cell Number Is Altered in a Cortical Layer-Specific Manner in Autism. Autism.

[52] Kim Y.S., Woo J., Lee C.J., Yoon B.E. (2017). Decreased Glial GABA and Tonic Inhibition in Cerebellum of Mouse Model for Attention-Deficit/ Hyperactivity Disorder (ADHD). Exp. Neurobiol..

